# Supplementary material for: Metamorphosis of memory circuits in Drosophila reveals a strategy for evolving a larval brain
Source: eLife. 2023 Jan 25;12:e80594. doi: 10.7554/eLife.80594 (PMC9984194; doi:10.7554/eLife.80594)
Supplement: Figure 2—source data 2. [file elife-80594-fig2-data2.pptx]

## Slide 1
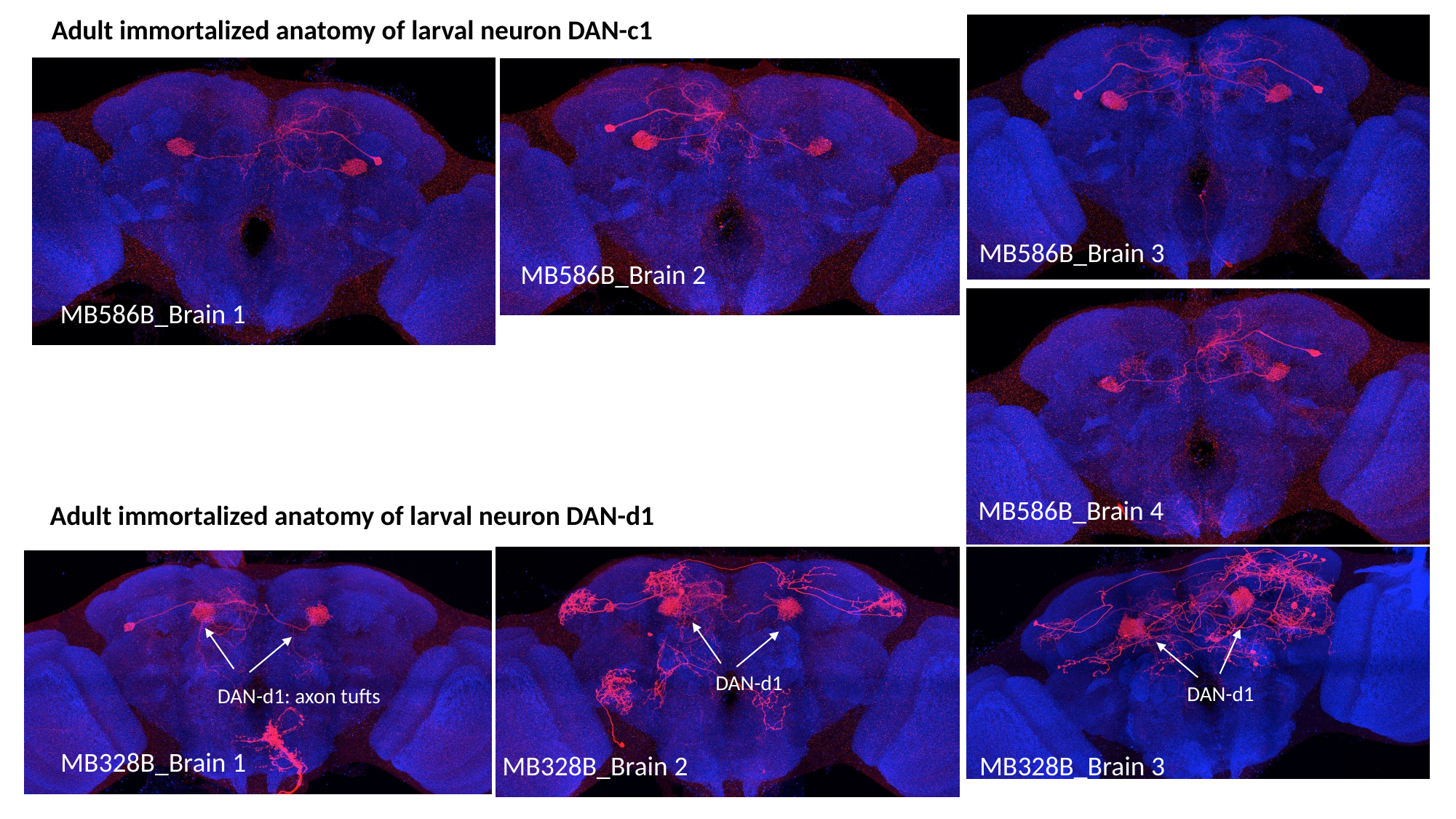

Adult immortalized anatomy of larval neuron DAN-c1
MB586B_Brain 3
MB586B_Brain 2
MB586B_Brain 1
MB586B_Brain 4
Adult immortalized anatomy of larval neuron DAN-d1
DAN-d1
DAN-d1
DAN-d1: axon tufts
MB328B_Brain 1
MB328B_Brain 2
MB328B_Brain 3
