# Supplementary material for: Metamorphosis of memory circuits in Drosophila reveals a strategy for evolving a larval brain
Source: eLife. 2023 Jan 25;12:e80594. doi: 10.7554/eLife.80594 (PMC9984194; doi:10.7554/eLife.80594)
Supplement: Figure 2—source data 3. [file elife-80594-fig2-data3.pptx]

## Slide 1
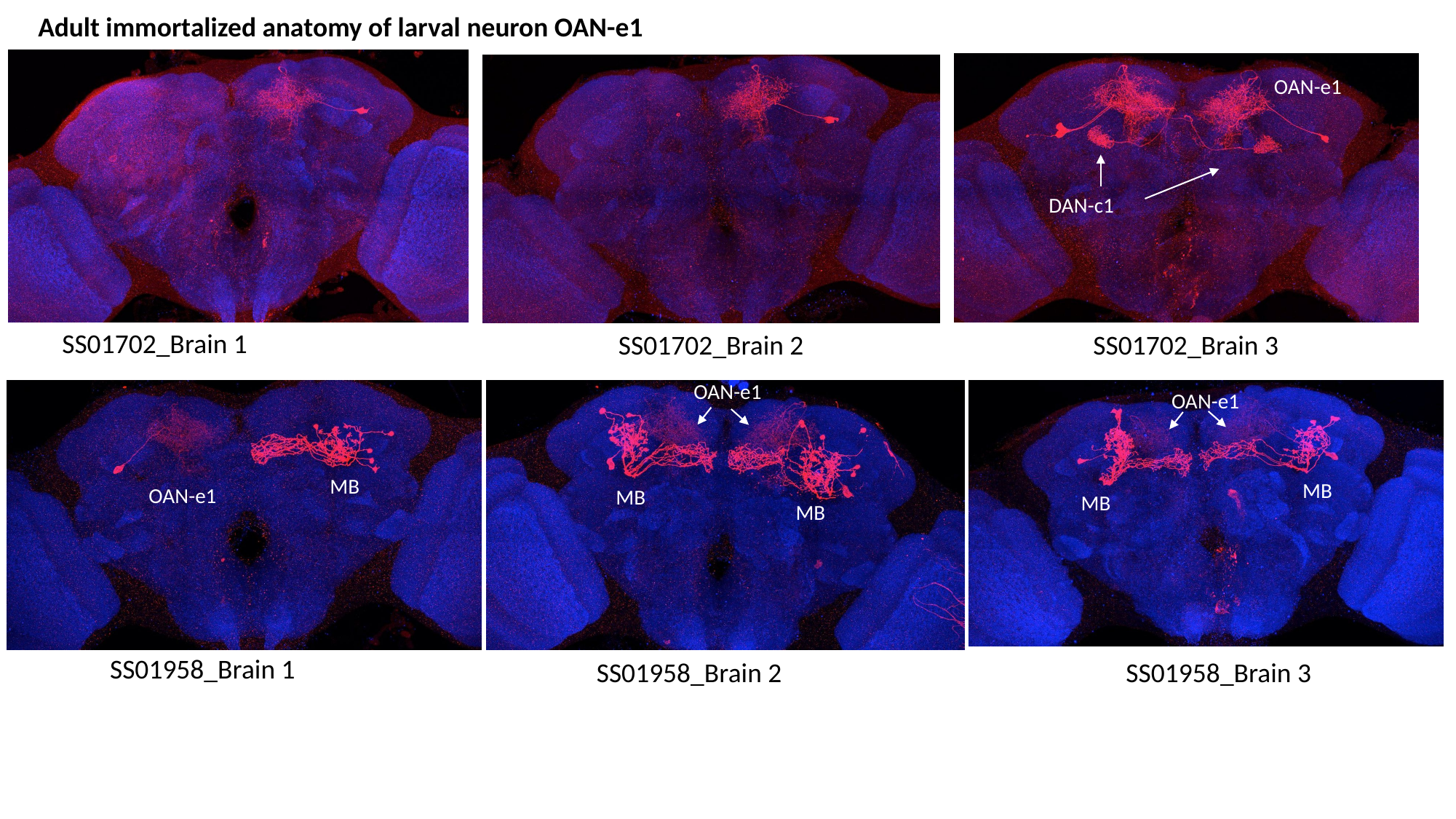

Adult immortalized anatomy of larval neuron OAN-e1
OAN-e1
DAN-c1
SS01702_Brain 1
SS01702_Brain 2
SS01702_Brain 3
OAN-e1
OAN-e1
MB
MB
OAN-e1
MB
MB
MB
SS01958_Brain 1
SS01958_Brain 2
SS01958_Brain 3
