# Supplementary material for: Metamorphosis of memory circuits in Drosophila reveals a strategy for evolving a larval brain
Source: eLife. 2023 Jan 25;12:e80594. doi: 10.7554/eLife.80594 (PMC9984194; doi:10.7554/eLife.80594)
Supplement: Figure 2—source data 4. — The anatomy of the adult form of MBIN-l1 was revealed using lines SS04484 and SS01624; that of DAN-f1 using lines MB065B and MB145B. [file elife-80594-fig2-data4.pptx]

## Slide 1
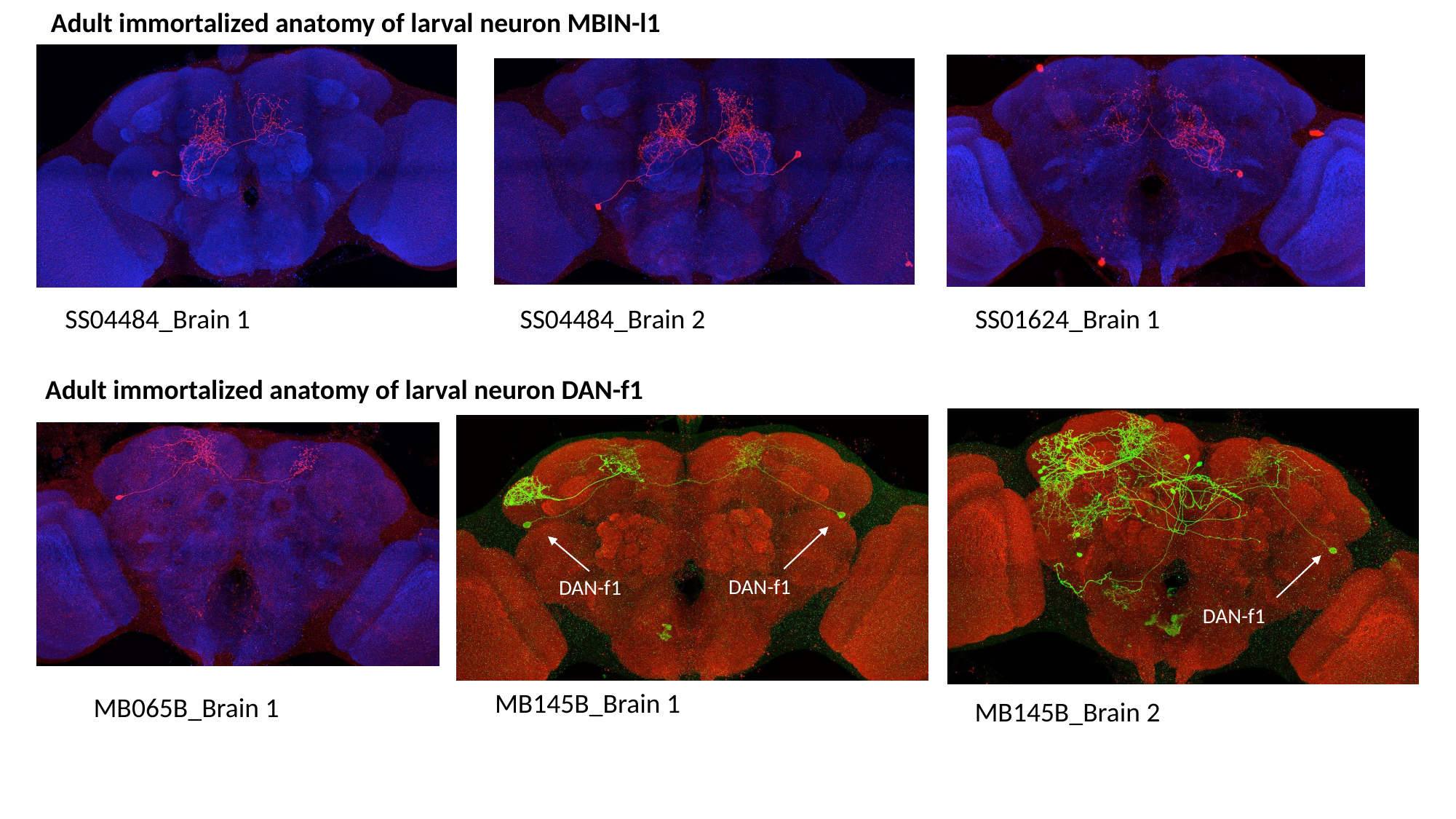

Adult immortalized anatomy of larval neuron MBIN-l1
DAN-d1
DAN-d1: axon tufts
SS04484_Brain 1
SS04484_Brain 2
SS01624_Brain 1
Adult immortalized anatomy of larval neuron DAN-f1
DAN-f1
DAN-f1
DAN-f1
MB145B_Brain 1
MB065B_Brain 1
MB145B_Brain 2
