# Supplementary material for: Metamorphosis of memory circuits in Drosophila reveals a strategy for evolving a larval brain
Source: eLife. 2023 Jan 25;12:e80594. doi: 10.7554/eLife.80594 (PMC9984194; doi:10.7554/eLife.80594)
Supplement: Figure 2—source data 5. — The anatomy of the adult form of DAN-g1 was revealed using lines SS017164 and SS01755; that of OAN-g1 using lines SS20844 and SS4268. [file elife-80594-fig2-data5.pptx]

## Slide 1
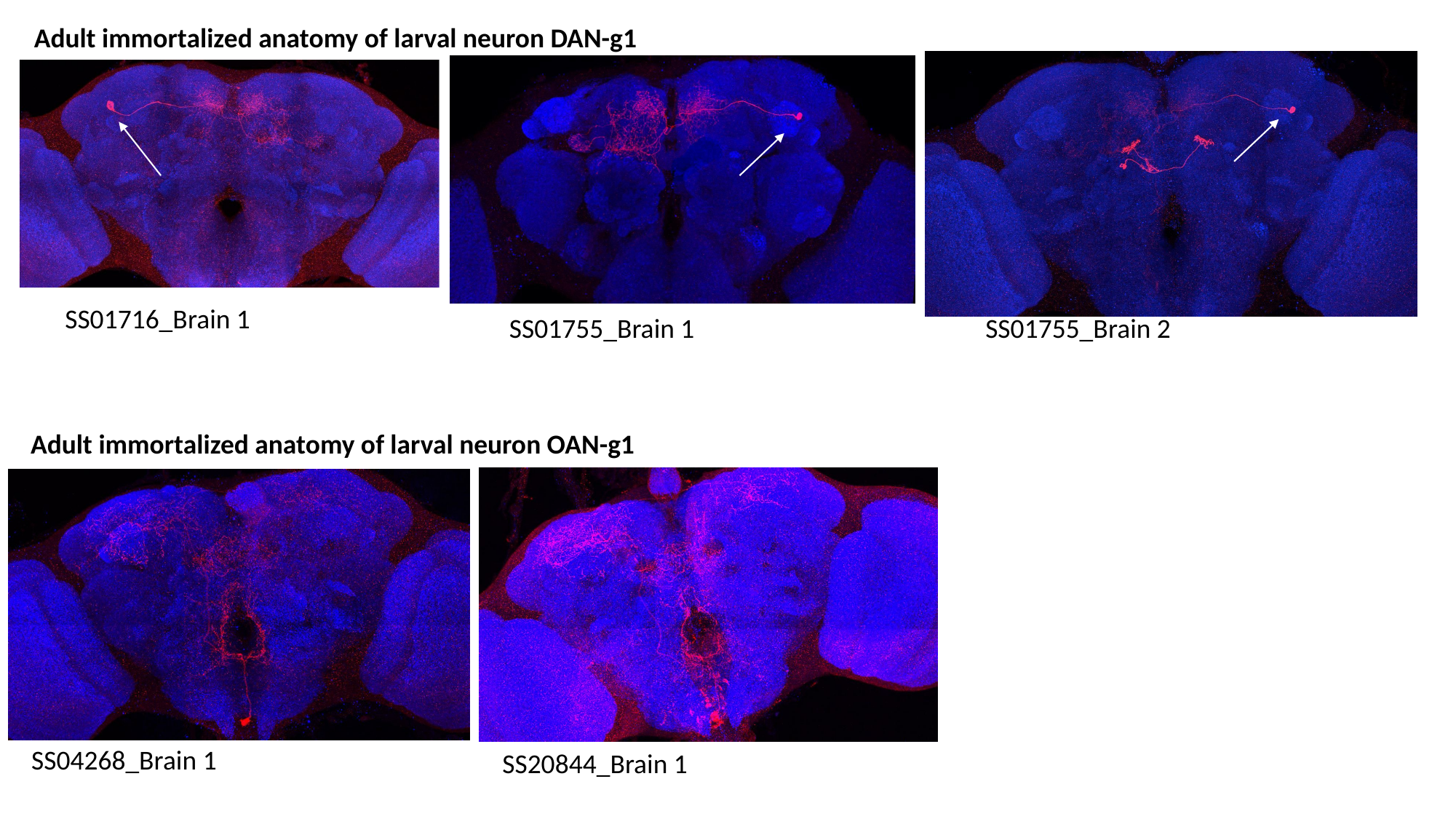

Adult immortalized anatomy of larval neuron DAN-g1
DAN-d1
DAN-d1: axon tufts
SS01716_Brain 1
SS01755_Brain 1
SS01755_Brain 2
Adult immortalized anatomy of larval neuron OAN-g1
SS04268_Brain 1
SS20844_Brain 1
