# Supplementary material for: Metamorphosis of memory circuits in Drosophila reveals a strategy for evolving a larval brain
Source: eLife. 2023 Jan 25;12:e80594. doi: 10.7554/eLife.80594 (PMC9984194; doi:10.7554/eLife.80594)
Supplement: Figure 3—source data 1. — The first line also revealed an occasional adult form of MBON-a2. [file elife-80594-fig3-data1.pptx]

## Slide 1
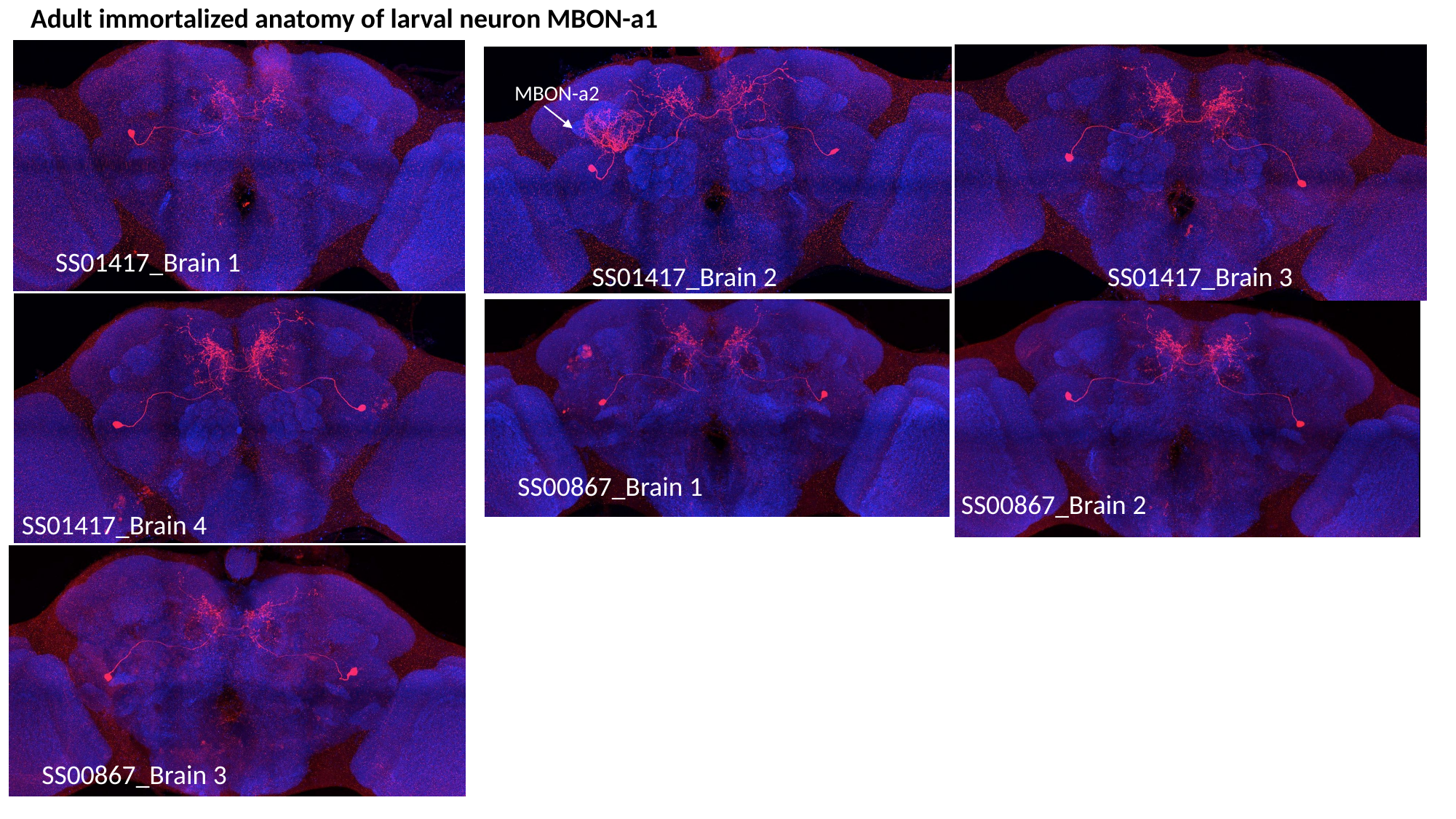

Adult immortalized anatomy of larval neuron MBON-a1
MBON-a2
DAN-d1
DAN-d1: axon tufts
SS01417_Brain 1
SS01417_Brain 2
SS01417_Brain 3
SS00867_Brain 1
SS00867_Brain 2
SS01417_Brain 4
SS00867_Brain 3
