# Supplementary material for: Metamorphosis of memory circuits in Drosophila reveals a strategy for evolving a larval brain
Source: eLife. 2023 Jan 25;12:e80594. doi: 10.7554/eLife.80594 (PMC9984194; doi:10.7554/eLife.80594)
Supplement: Figure 3—source data 2. — The anatomy of the adult form of MBON-a2 was revealed using lines SS00872 and SS02006; that of MBON-b1,-b2 using lines SS01708 and SS01959. [file elife-80594-fig3-data2.pptx]

## Slide 1
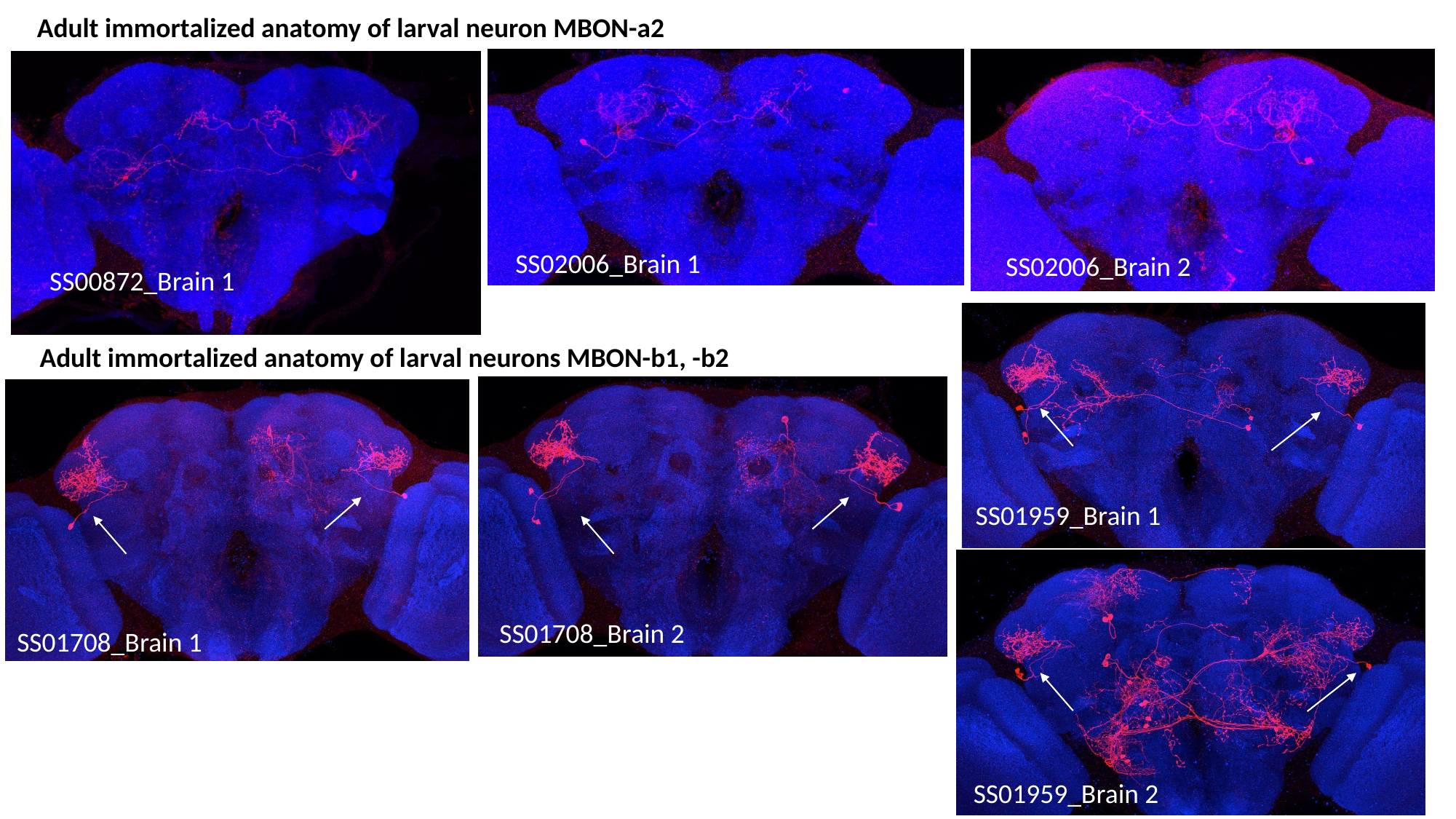

Adult immortalized anatomy of larval neuron MBON-a2
SS02006_Brain 1
SS02006_Brain 2
SS00872_Brain 1
Adult immortalized anatomy of larval neurons MBON-b1, -b2
SS01959_Brain 1
SS01708_Brain 2
SS01708_Brain 1
SS01959_Brain 2
