# Supplementary material for: Metamorphosis of memory circuits in Drosophila reveals a strategy for evolving a larval brain
Source: eLife. 2023 Jan 25;12:e80594. doi: 10.7554/eLife.80594 (PMC9984194; doi:10.7554/eLife.80594)
Supplement: Figure 3—source data 3. — The anatomy of the adult form of the three neurons was revealed using lines SS01705, SS04172, and SS04328, respectively. [file elife-80594-fig3-data3.pptx]

## Slide 1
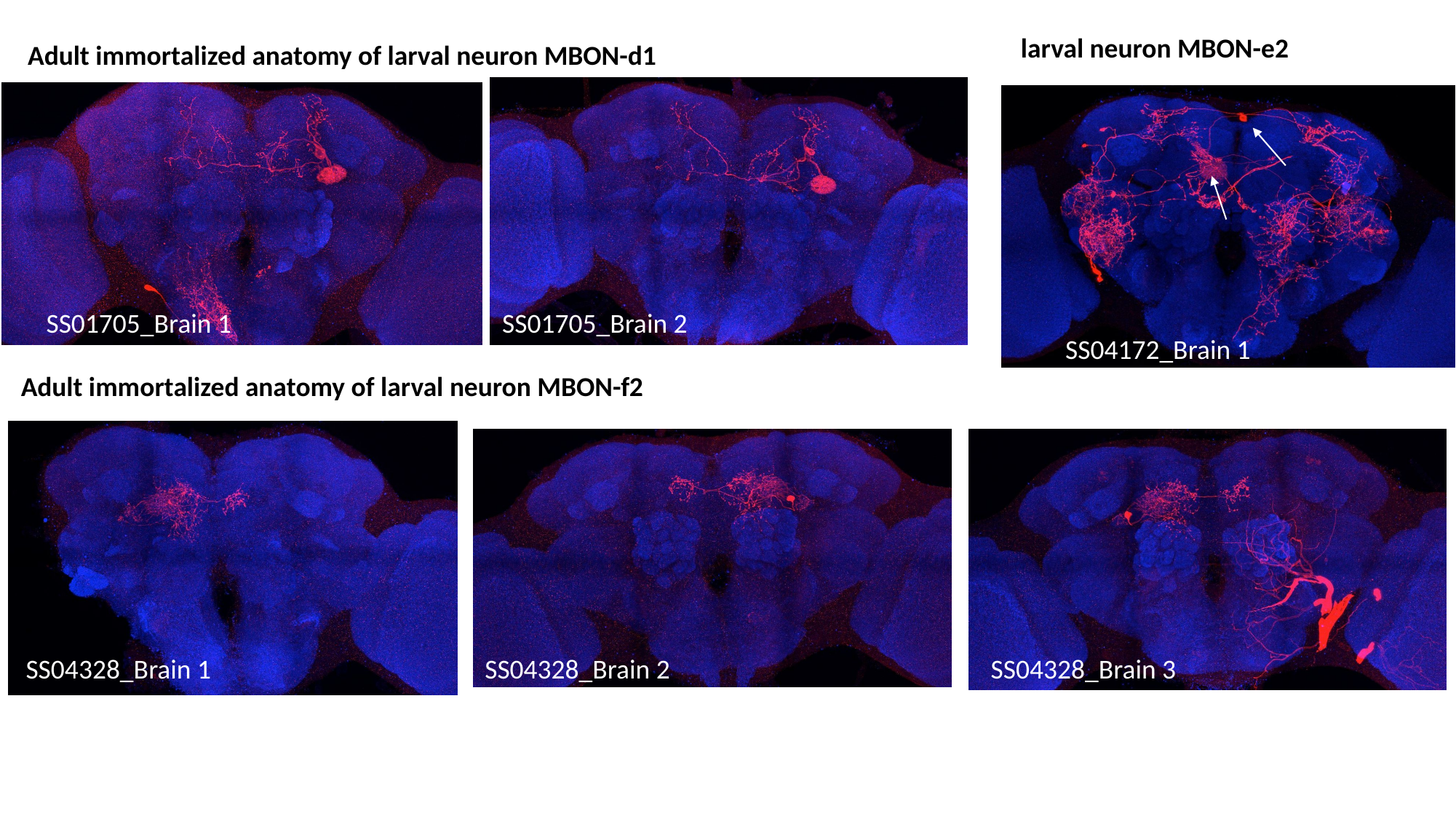

larval neuron MBON-e2
Adult immortalized anatomy of larval neuron MBON-d1
SS01705_Brain 1
SS01705_Brain 2
SS04172_Brain 1
Adult immortalized anatomy of larval neuron MBON-f2
SS04172_Brain 1
SS04328_Brain 1
SS04328_Brain 2
SS04328_Brain 3
