# Supplementary material for: Metamorphosis of memory circuits in Drosophila reveals a strategy for evolving a larval brain
Source: eLife. 2023 Jan 25;12:e80594. doi: 10.7554/eLife.80594 (PMC9984194; doi:10.7554/eLife.80594)
Supplement: Figure 3—source data 4. [file elife-80594-fig3-data4.pptx]

## Slide 1
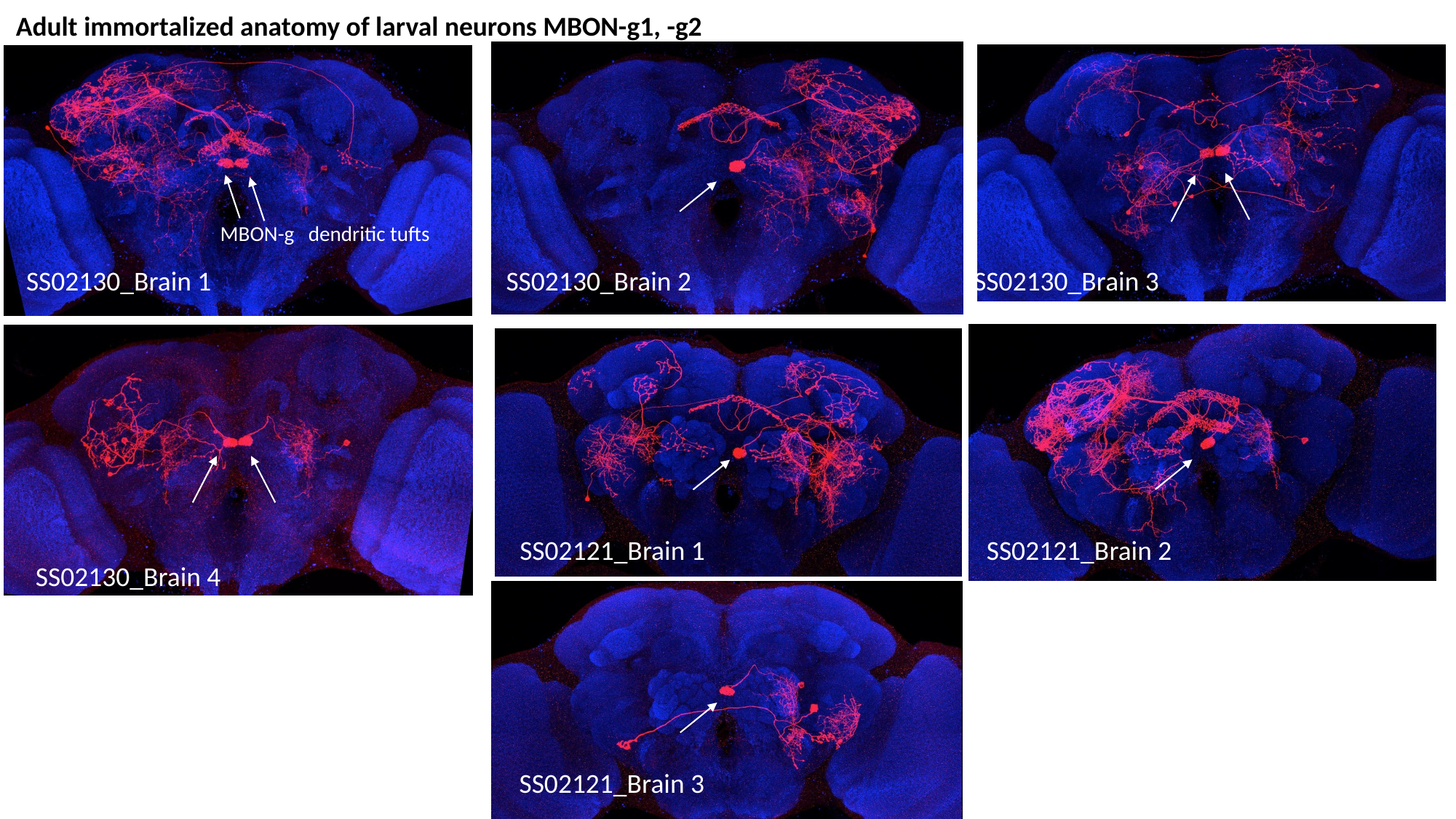

Adult immortalized anatomy of larval neurons MBON-g1, -g2
MBON-g dendritic tufts
SS02130_Brain 1
SS02130_Brain 1
SS02130_Brain 2
SS02130_Brain 3
SS02121_Brain 1
SS02121_Brain 2
SS02130_Brain 4
SS02121_Brain 3
