# Supplementary figures and images for: Metamorphosis of memory circuits in Drosophila reveals a strategy for evolving a larval brain
Source: eLife. 2023 Jan 25;12:e80594. doi: 10.7554/eLife.80594 (PMC9984194; doi:10.7554/eLife.80594)

## Slide 1
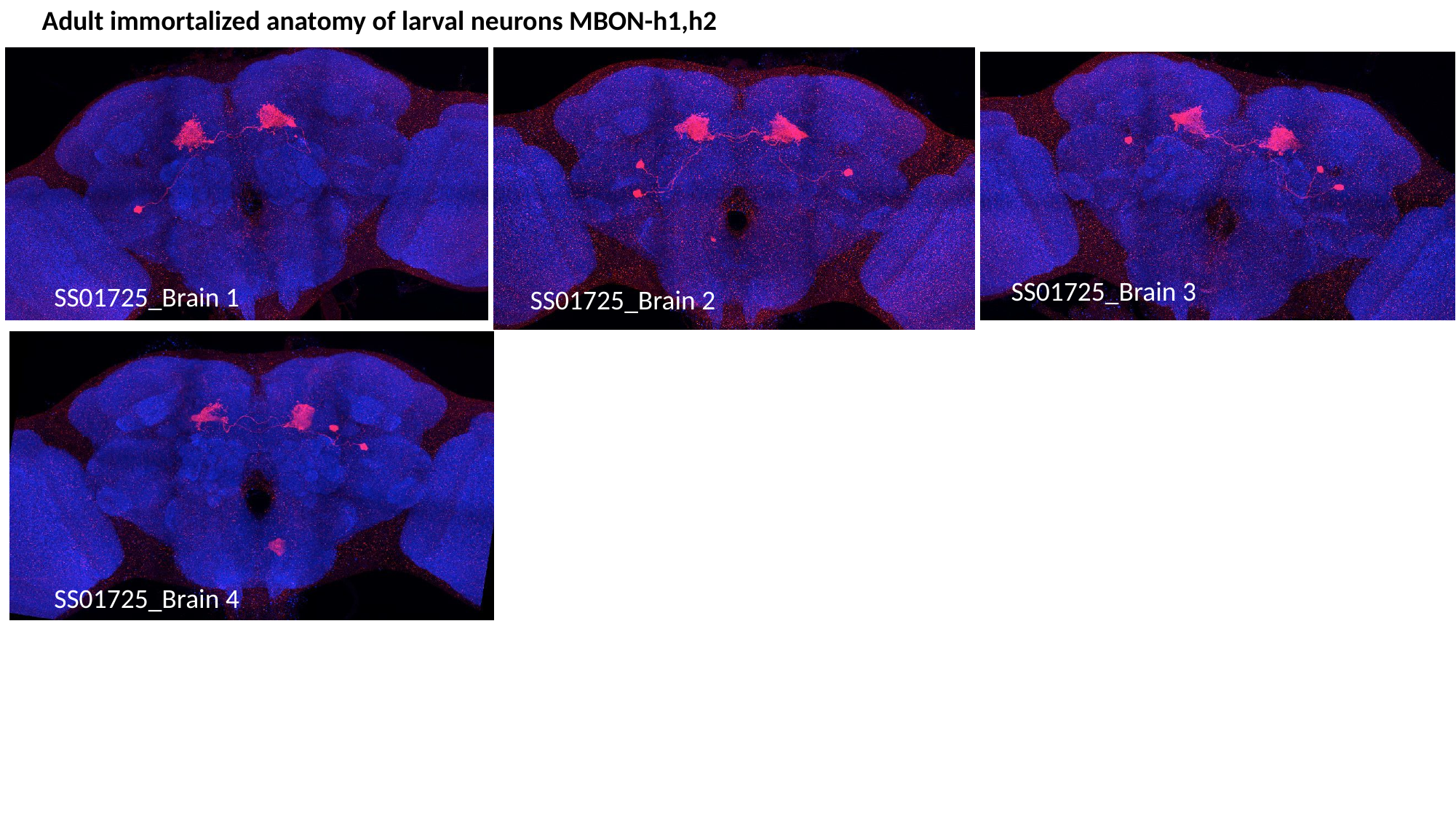

Adult immortalized anatomy of larval neurons MBON-h1,h2
SS01725_Brain 3
SS01725_Brain 1
SS01725_Brain 2
SS01725_Brain 4

Supplement: Figure 3—source data 5. [file elife-80594-fig3-data5.pptx]
