# Supplementary material for: Metamorphosis of memory circuits in Drosophila reveals a strategy for evolving a larval brain
Source: eLife. 2023 Jan 25;12:e80594. doi: 10.7554/eLife.80594 (PMC9984194; doi:10.7554/eLife.80594)
Supplement: Figure 3—source data 6. [file elife-80594-fig3-data6.pptx]

## Slide 1
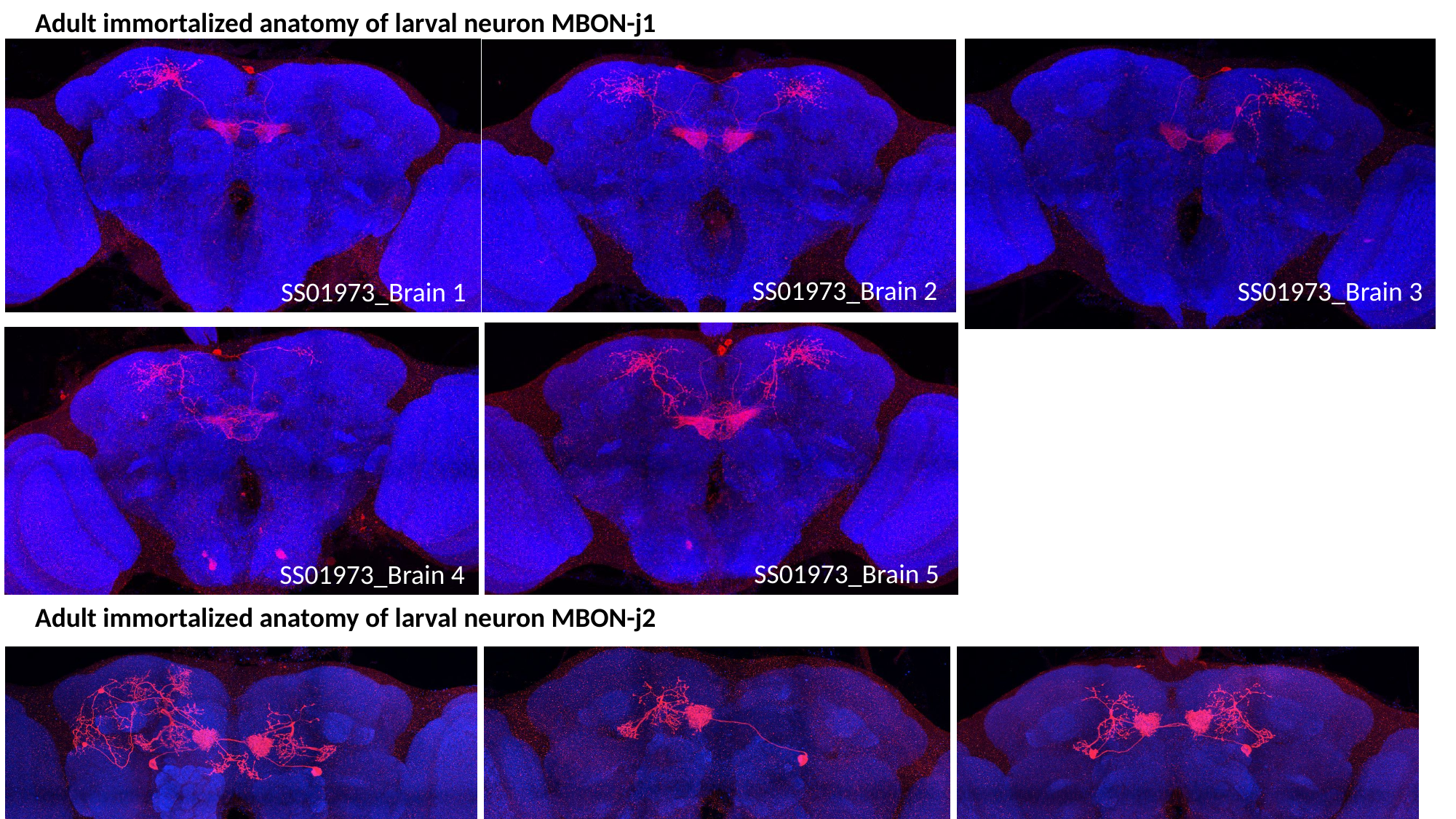

Adult immortalized anatomy of larval neuron MBON-j1
SS01973_Brain 2
SS01973_Brain 3
SS01973_Brain 1
SS01973_Brain 5
SS01973_Brain 4
Adult immortalized anatomy of larval neuron MBON-j2
SS00860_Brain 3
SS00860_Brain 1
SS00860_Brain 2
