# Supplementary material for: Metamorphosis of memory circuits in Drosophila reveals a strategy for evolving a larval brain
Source: eLife. 2023 Jan 25;12:e80594. doi: 10.7554/eLife.80594 (PMC9984194; doi:10.7554/eLife.80594)
Supplement: Figure 3—source data 7. [file elife-80594-fig3-data7.pptx]

## Slide 1
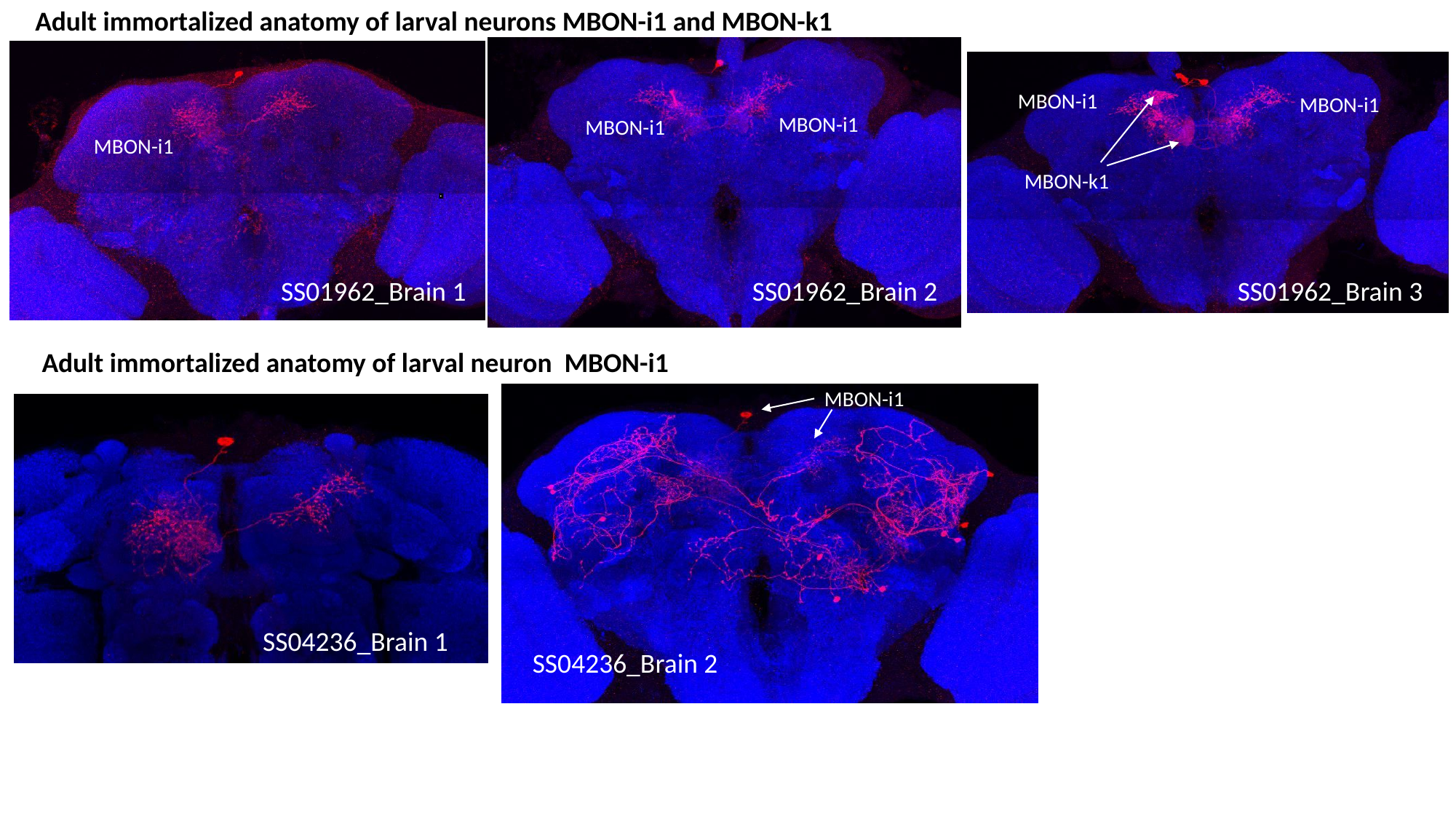

Adult immortalized anatomy of larval neurons MBON-i1 and MBON-k1
MBON-i1
MBON-i1
MBON-i1
MBON-i1
MBON-i1
MBON-k1
SS01962_Brain 1
SS01962_Brain 2
SS01962_Brain 3
Adult immortalized anatomy of larval neuron MBON-i1
MBON-i1
SS04236_Brain 1
SS04236_Brain 2
