# Supplementary material for: Metamorphosis of memory circuits in Drosophila reveals a strategy for evolving a larval brain
Source: eLife. 2023 Jan 25;12:e80594. doi: 10.7554/eLife.80594 (PMC9984194; doi:10.7554/eLife.80594)
Supplement: Figure 4—source data 1. [file elife-80594-fig4-data1.pptx]

## Slide 1
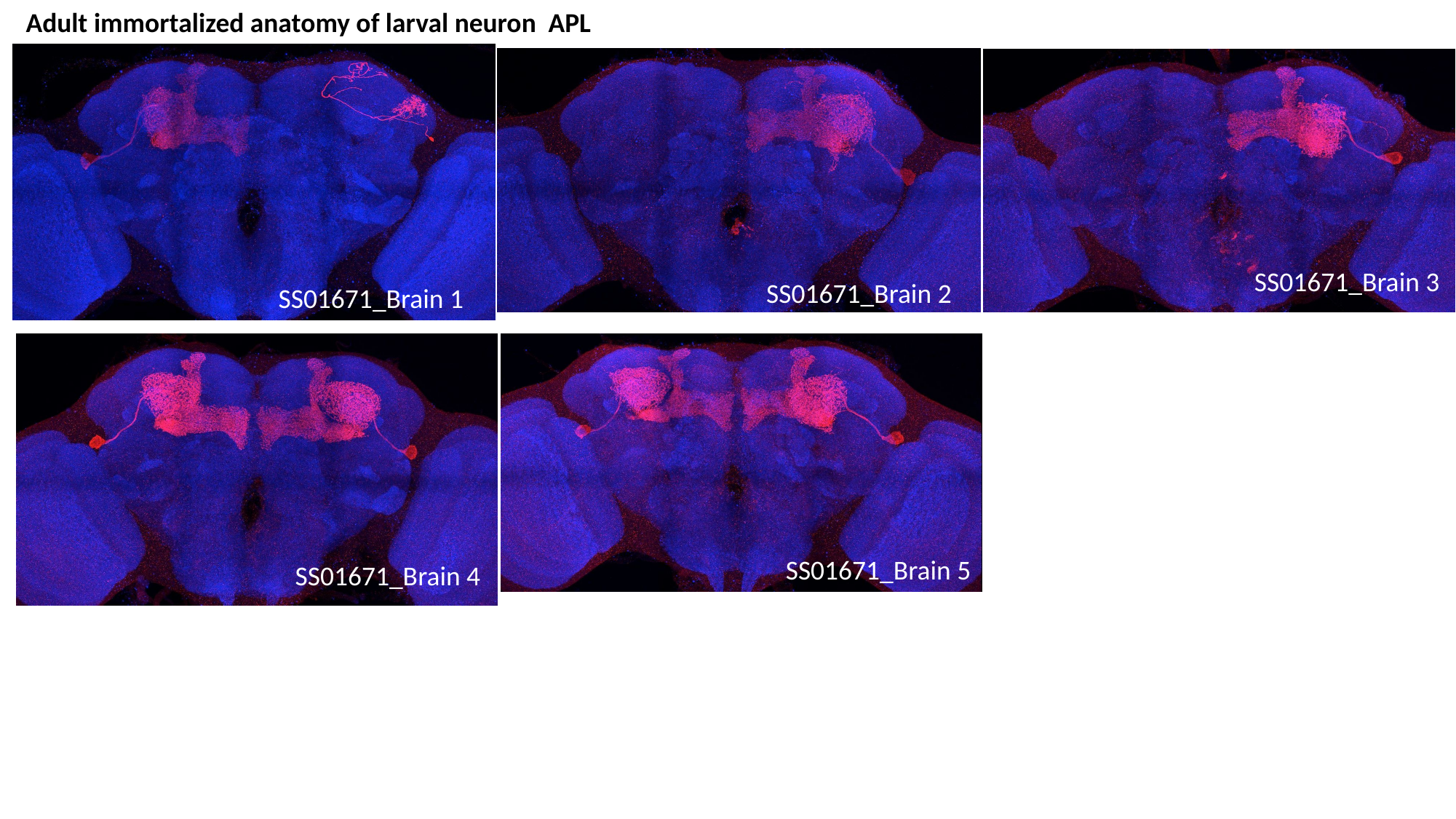

Adult immortalized anatomy of larval neuron APL
MBON-i1
SS01671_Brain 3
SS01962_Brain 1
SS01671_Brain 2
SS01671_Brain 1
SS01671_Brain 5
SS01671_Brain 4
